# The synergistic role of gut microbiota and RNA in metabolic diseases: mechanisms and therapeutic insights

**DOI:** 10.3389/fmicb.2025.1504395

**Published:** 2025-01-29

**Authors:** Zhuo Huang, Qinyan Yao, Shuang Ma, Jinjie Zhou, Xiaoxuan Wang, Qingguo Meng, Yaxin Liu, Zihan Yu, Xin Chen

**Affiliations:** ^1^Department of Gastroenterology and Hepatology, Tianjin Medical University General Hospital, Tianjin, China; ^2^Tianjin Institute of Digestive Disease, Tianjin Medical University General Hospital, Tianjin, China

**Keywords:** RNA, metabolic diseases, gut microbiota, metabolism, microbial metabolites

## Abstract

The gut microbiota plays a pivotal role in human metabolic health by influencing immune responses, digestion, and metabolic homeostasis. Recent research highlights the intricate interactions between gut microbiota and RNA, especially non-coding RNAs, in regulating metabolic processes. Dysbiosis of the gut microbiota has been linked to metabolic disorders such as type 2 diabetes, obesity, metabolic-associated fatty liver disease (MAFLD) and metabolic heart disease. Microbial metabolites, including short-chain fatty acids (SCFAs), modulate RNA expression, influencing lipid metabolism, glucose regulation, and inflammatory responses. Additionally, microRNAs (miRNAs) and long non-coding RNAs (lncRNAs) serve as critical regulators in these processes, with emerging evidence showing that gut-derived metabolites affect post-transcriptional gene regulation. This review synthesizes the current understanding of the gut microbiota-RNA axis and its role in metabolic diseases. By exploring the molecular mechanisms, particularly how gut microbiota-derived signals modulate RNA pathways, the review underscores the potential of targeting this axis for therapeutic interventions. Furthermore, it examines how dysbiosis leads to epigenetic changes such as m6A RNA methylation, contributing to disease pathogenesis. These insights offer a new perspective on the prevention and treatment of metabolic diseases, with potential applications in personalized medicine.

## Introduction

Current epidemiological, pathological, and histological studies at both cellular and animal levels reveal that gut microbiota significantly influence metabolic health and disease risk in humans (Fardi et al., 2023). These microbial communities, which colonize various luminal surfaces of the body, encompass vast numbers of bacteria, fungi, phages, eukaryotic viruses, and other microorganisms, most of which are symbiotic or mutualistic (Carding et al., 2015; Jandhyala et al., 2015; Koutoukidis et al., 2024; Thursby and Juge, 2017). The collective gut microbial genes of an individual represent a gene pool far larger than that of the human genome. The gut microbiota plays a critical role in enhancing host immunity, digestion, endocrine and neural signaling, drug metabolism, endotoxin clearance, and regulating the production of metabolites essential to host health (Flint et al., 2012).

In the past two decades, evidence from observational studies has established a robust link between gut microbiota and host metabolic homeostasis, suggesting that dysbiosis of the gut microbiota is a contributing factor in the pathogenesis of several prevalent metabolic disorders, including type 2 diabetes, obesity, MAFLD and metabolic heart disease (Icaza-Chávez, 2013). For example, the fermentation of dietary fiber by gut microbiota, particularly from the phyla Firmicutes and Bacteroidetes, produces SCFAs such as butyrate, propionate, and acetate, which modulate host metabolism via G-protein-coupled receptors (GPCRs) expressed by intestinal endocrine cells (Scheppach, 1994). Acetate and butyrate stimulate the release of glucagon-like peptide 1 (GLP-1) and peptide YY (PYY), influencing pancreatic insulin biosynthesis (GLP-1 induced) and central appetite regulation (PYY induced satiety) (Drucker, 2022; Pittner et al., 2004; Scheppach, 1994).

RNA serves as a key mediator in the transmission of genetic information and is classified into several major types. Small RNAs such as miRNAs and small interfering RNAs (siRNAs) are essential for regulating gene expression at the post-transcriptional level (Li et al., 2008; Lee et al., 2004). lncRNAs exert significant influence on gene regulation by modulating chromatin architecture, transcription, and translation processes. In the study of metabolic diseases, the expression profile and functional regulation of RNA molecules have become an important research direction (Decatur and Fournier, 2002; Kong et al., 2018; Zhao et al., 2017). Recently, a growing number of researches have focused on the relationship between RNA and gut microbiota in metabolic disorders, for example, The main RNAs associated with metabolic heart disease are miR-21, miR-126, miR-33, lncRNA-H19, lncRNA-H19, lncRNA-ANRIL (Krichevsky and Gabriely, 2009; Olivo-Martínez et al., 2024; Surina et al., 2021).

The intestinal metabolism of phytoestrogens is also associated with metabolic diseases and their amelioration. The gut microbiota metabolizes phytoestrogens, such as isoflavones, ellagic acid derivatives, and lignans, into more bioactive metabolites, including equol, urolithins, and enterolignans. These metabolites, due to their higher lipophilicity and enhanced bioavailability, may exhibit stronger biological activity compared to their precursor compounds. These metabolites can interact with key enzymes involved in cellular processes, reducing DNA damage and lipid peroxidation (Stojanov and Kreft, 2020).

Although most existing reviews focus on the relationship between metabolic diseases and the gut microbiota—primarily highlighting strain abundance or metabolites affecting host receptors and contributing to metabolic disorders, or separately examining the role of RNA in metabolic diseases—limited attention has been given to the integrated effects of gut microbiota and its derivatives with RNA on metabolic diseases. Mechanistic studies in this area are critical for advancing future research. To address this gap, this review synthesizes the intricate interactions between metabolic diseases, gut microbiota, and RNA, with an emphasis on the molecular mechanisms through which gut microbiota modulates host metabolism via RNA pathways. It further examines the specific roles and manifestations of these RNA-mediated mechanisms in the context of metabolic disorders. Through a comprehensive analysis of current research, we aim to offer new insights and directions for future studies, while providing a scientific foundation for the prevention and treatment of metabolic diseases.

## Gut microbiota regulates host RNA trigger

Recent studies have revealed that the influence of the gut microbiota extends even to the molecular level, where it can finely regulate the host’s RNA expression, thereby affecting gene function and cellular behavior (Chiba et al., 2024; Wortelboer et al., 2022).

In addition, it was found that gut microbiota produces a short-chain fatty acid butyrate that targets the DNA methylation level of the host miR-378a promoter, thus preventing the development of obesity and glucose intolerance (Krist et al., 2015). Second, in 2019, Virtue et al. found that indole-3-carboxylic acid produced by gut microbes controlled miR-181 family expression in mouse white adipocytes, further negatively regulating energy expenditure and insulin sensitivity (Virtue et al., 2019). This suggests that inhibitory regulation of miRNAs by gut microbe-derived metabolites is the central mechanism. Intestinal flora can affect miRNA expression in intestinal epithelial cells (IEC). Nakata et al. found that gut microbiota induced miR-21-5p expression in IEC, and mmu-miR-21-5p increased intestinal epithelial permeability by down-regulating the expression of ADP ribosylation factor 4 (arf4) in 2017 (Nakata et al., 2017). The above studies suggest that miRNAs may act as intermediate mediators to mediate the regulation of host IEC gene expression by gut microbiota.

When the organism is immunocompromised, *pks+ E. coli* containing pks gene islands, *E. coli* can colonize the gut and induce disease. It has been found that the genotoxin Colibactin produced by *pks+ E. coli* activates the c-Myc pathway by disrupting the DNA double strand, resulting in an increase in downstream miR-20a-5p expression (Cao et al., 2024; Sun et al., 2024).

The miRNAs are partially involved in mediating the expression of miR-20a-5p in *E. coli*. This suggests that miRNAs are partially involved in mediating the pathogenic effects of *pks+ E. coli* (Huang et al., 2022). The mechanism through which the gut microbiota regulates miRNA expression is still not fully understood. Is it through the direct regulation of miRNA expression by the host or its cells, or through the indirect regulation of miRNA expression by the metabolites in the intestinal tract? The specific mechanism remains to be studied in depth.

## RNA regulates the homeostasis of gut microbiota

RNA molecules, particularly miRNAs, play a crucial role in maintaining the homeostasis of the gut microbiota. Recent studies have shown that RNA not only plays a key role in host cell proliferation, differentiation, and immune responses but also directly influences the health status of the host by regulating the composition and function of the gut microbiota (Monzel and Desai, 2024). Specifically, certain miRNAs can indirectly affect the composition of the gut microbiota through receptors or secreted substances in host cells. For example, the bacterium *Segatella copri* is a common member of the human gut microbiota that is associated with both health and disease states. Through extensive transcriptomic analysis of *S. copri* and human-derived samples, researchers identified and named the *S. copri* RNA colonization factor (SrcF). Deletion of the SrcF gene significantly affects the ability of *S. copri* to colonize the gut and disrupts the expression of genes involved in nutrient uptake and utilization, including PULs. SrcF may represent a regulatory hub that integrates nutrient signals essential for *S. copri* survival in the gut (El Mouali et al., 2024).

Mechanistic research in this area is still limited, with most studies focusing on the exosome perspective. Exosomes are secreted by almost all cell types via exocytosis and establish cross-talk between the host and the gut microbiota. The major components of exosomes include miRNAs. miRNAs that influence microbial growth in the gut are primarily derived from IECs and homeodomain-only protein homeobox (HOPX)-positive cells. In colitis-tolerant mice, stimulation with DSS induces IECs to secrete miR-142a-3p, which specifically promotes the growth of *Lactobacillus reuteri* by binding to specific targets in the bacterial genome (Zhang et al., 2023). On the other hand, miR-200b-3p, miR-200b-5p, and miR-26a-5p are negatively correlated with *Dubosiella* and *Lactobacillus* but positively correlated with *Escherichia-Shigella*. The improved microbiota, supplemented with miR-200b-3p, can feedback to the gut microenvironment via exosomes, thus inhibiting the development of intestinal inflammation (He et al., 2022).

Furthermore, RNA molecules also regulate the metabolic environment, and antimicrobial peptide secretion in the gut, directly influencing the growth and proliferation of specific microbial species (Liu et al., 2016). For instance, by culturing the anaerobic bacterium *Fusobacterium nucleatum* (Fn) and *E. coli* with synthesized miRNA mimics *in vitro*, it was found that hsa-miR-515-5p promotes the growth of *Fn*, while hsa-miR-1226-5p promotes the growth of *E. coli*. These findings indicate that miRNAs directly influence bacterial growth (Shen et al., 2022). Through these mechanisms, RNA molecules participate in the fine-tuned regulation of the gut microbiota, thereby affecting the host’s immune responses, metabolic functions, and even disease susceptibility.

## RNA, gut microbiota, and diabetes

With changes in modern lifestyles, the incidence of metabolic disease, such as diabetes has significantly increased (de Lima et al., 2024). This condition is complex, having long disease course, and is difficult to cure. The pathological processes involve all aspects of life activities, including epigenetics, proteomics, and metabolomics (Suhre and Zaghlool, 2021). Current studies suggest that the mutual regulatory effects between RNA and the gut microbiota play an irreplaceable role in controlling host metabolism, and their imbalance is likely to contribute to the onset of related metabolic diseases (Mundula et al., 2022; Nie et al., 2019). Further analysis may provide important indicators for the early prediction of metabolic diseases like diabetes and help define future personalized therapeutic targets.

Diabetes includes type 1 diabetes (T1DM), characterized by pancreatic β-cell dysfunction and absolute insulin deficiency, and type 2 diabetes (T2DM), where β-cells have not completely lost the ability to produce insulin, resulting in relative insulin deficiency or resistance (Argano et al., 2023; dos Santos Haber et al., 2023). RNA is considered a crucial determinant of pancreatic β-cell functional integrity, as it regulates β-cell development and dysfunction, controls insulin signaling in peripheral tissues like adipose tissue to maintain glucose homeostasis, and is involved in β-cell proliferation, apoptosis, insulin biosynthesis, and secretion (Benáková et al., 2021; Gao et al., 2022). To date, numerous diabetes-related RNAs have been identified, including miR-21, miR-424-5p, circ-HIPK, lncRNA-MALAT1, circRNA-HIPK3 and miR-30a (Alrefai et al., 2023; Fu et al., 2014; Grieco et al., 2017; Lakhter et al., 2018).

Specifically, the upregulation of miR-21 inhibits the expression of the anti-apoptotic gene Bcl-2, leading to a reduction in Bcl-2 protein synthesis, while simultaneously increasing the pro-apoptotic protein caspase-3, thereby inducing β-cell apoptosis. This results in decreased insulin secretion and elevated blood glucose levels (Nigi et al., 2018; Shi et al., 2010; Ye et al., 2018). miR-424-5p directly interacts with the 3’ UTR sequence of insulin receptor (INSR) mRNA in human hepatocellular carcinoma cell lines, thereby suppressing insulin gene expression and signaling in adipocytes. Additionally, RNA has been implicated in the pathogenesis of diabetic retinopathy. lncRNA-MALAT1 has been shown to promote inflammation and fibrosis in diabetic retinopathy by activating the NF-κB and TGF-β1 signaling pathways (Abedi et al., 2024; Jia and Zhou, 2020; Li et al., 2021; Ren and Wang, 2021). Similarly, circRNA-HIPK3 promotes endothelial cell proliferation and vascular dysfunction in diabetic retinopathy by blocking the function of miR-30a (Pan et al., 2018; Zaiou, 2020; Zhou and Kuang, 2021).

At the same time, an increasing number of studies suggest that the gut microbiota plays an essential role in the development of diabetes driven by RNA (Tanase et al., 2020). In both T1DM and T2DM patients, the abundance and diversity of the gut microbiota are altered to varying degrees. A meta-analysis by Qin et al. on 345 Chinese T2DM patients revealed a decrease in butyrate-producing gut bacteria, such as *Clostridium* and *Butyricicoccus*, while the abundance of opportunistic pathogens increased in 2024 (Wu et al., 2024). The gut microbiota assists the host in metabolizing and breaking down dietary intake, converting indigestible polysaccharides in food into SCFAs. The SCFA pathways of the gut microbiota not only provide energy for the host but also maintain the metabolic balance of unbound lipids and glucose in tissues through the activation of their receptor, G protein-coupled receptor 41 (GPR41/43) (Wu et al., 2021). This process interferes with insulin signaling in fat cells, reducing fat accumulation in adipose tissue.

SCFAs, including butyrate, acetate, and propionate, have diverse functions, with butyrate specifically recognized for its antioxidant properties and its ability to suppress autoimmune reactions associated with diabetes (Rasouli-Saravani et al., 2023). Research has shown that butyrate expression is negatively correlated with miR-106a, and reduced miR-106a expression increases the expression of the cell cycle inhibitor p21, partially inhibiting the proliferation of tumor cells. Additionally, miR-21, which was previously mentioned for directly acting on the target gene Bcl-2 to induce β-cell apoptosis, is also thought to be regulated by the gut microbiota during diabetes development (Gao and Zhao, 2020).

Researchers investigating metabolic diseases like obesity and diabetes have discovered that in high-fat diet-induced mouse models, the triglyceride levels in the liver are associated with both the gut microbiota and the expression of liver miRNAs (Wilson et al., 2017). This suggests that the interaction between the gut microbiota and liver miR-21 expression might mediate metabolic effects via the regulation of miR-21 target genes. Studies showed that germ-free mice had lower liver triglyceride levels compared to other models, and miR-18a, miR-666, and miR-21 were all regulated in wild-type mouse hepatocytes by the pro-inflammatory LPS released from *Escherichia coli* O55 in a LPS-dependent manner (Arunmanee et al., 2016).

Furthermore, liver triglyceride levels were positively correlated with the relative abundance of *Firmicutes*, and negatively correlated with the expression of liver miR-21 and the relative abundance of *Proteobacteria* and *Bacteroidetes* (Shin et al., 2015). The pathological mechanism underlying this process is likely similar to the regulation of miR-21-5p expression by the gut microbiota mentioned earlier. The gut microbiota can highly specifically regulate miR-21-5p through excessive secretion of LPS and peptidoglycan (PGN), activating Toll-like receptor 2/4 (TLR2/4)-related pathways, promoting the expression of pro-inflammatory cytokines and nitric oxide in antigen-presenting cells, and inducing NF-κB activation and interferon upregulation, which triggers an inflammatory immune response (Casado-Bedmar et al., 2024).

In a chronic inflammatory environment, macrophages release TNF-α, IL-1β and IL-18, which interfere with insulin signaling between the liver, fat, and other target tissues, ultimately leading to abnormal hepatocyte apoptosis, non-alcoholic fatty liver disease, insulin resistance, and metabolic endotoxemiat (Lee et al., 2023). Therefore, the composition and metabolic characteristics of the gut microbiota determine its critical role in diabetes onsetk (Cordeiro et al., 2024). Blocking the transmission of specific miRNA regulatory signals from gut microbial metabolites at certain targets may hold great significance in preventing or delaying diabetes-related complications and other metabolic disorders (Prattichizzo et al., 2021).

As the most common post-transcriptional epigenetic modification, m6A exhibits abnormal modification patterns in the β-cells of diabetic islets and in various diabetic complications, disrupting the expression of key target genes and thus influencing disease pathology (Eizirik et al., 2020). Therefore, both gut microbiota dysbiosis and abnormal m6A modification profiles at disease sites reflect the dynamic changes involved in the development of T2DM at the micro level (Benak et al., 2023). Furthermore, recent studies have proposed that gut microbiota, as an environmental factor, can directly or indirectly affect m6A methylation levels in the body, leading to the hypothesis of a gut microbiota-m6A metabolic axis. High-throughput sequencing has shown differences in m6A modification levels across various tissues and organs between germ-free mice and conventional mice, which are associated with changes in transcriptional levels (Jabs et al., 2020; Zhuo et al., 2022). For example, mouse models colonized with single strains like *Akkermansia muciniphila* and *Lactobacillus plantarum* exhibit specific alterations in m6A modifications (Guo et al., 2023). In 2022, Chen et al. further confirmed the causal role of gut microbiota in regulating m6A modifications through studies on both animal and human samples (Chen et al., 2023). They found that *Fusobacterium nucleatum* can activate the YAP signaling pathway, downregulating the transcription of METTL3, significantly reducing m6A modifications in intestinal epithelial tissues, and promoting the expression of serine/threonine-protein kinase 26B, thus enhancing the invasiveness of colorectal cancer (Wang et al., 2020). This indicates that changes in m6A modifications are a crucial mechanism by which the gut microbiota exerts physiological effects (D’Aquila et al., 2020).

Current research suggests two main pathways through which the gut microbiota influences host methylation modifications: direct and indirect pathways (Bhat and Kapila, 2017).

### Direct pathway

The gut microbiota can directly affect the chromatin and transcriptome states of intestinal cells through structural components such as flagellin, LPS, and peptidoglycan (Wu et al., 2022). Intestinal cells’ leukocyte antigens, major histocompatibility complex gene systems, and pattern recognition receptors can recognize and differentiate bacterial antigens, triggering corresponding responses and forming epigenetic memory (Kmieć et al., 2017). Studies have shown that LPS stimulation alters m6A methylation levels in the liver and activates the nucleotide-binding oligomerization domain (NOD) protein 1/NF-κB pathway, mediating liver inflammation and damage (Satheesan et al., 2024). Additionally, LPS stimulation leads to enhanced m6A modifications in the liver, associated with upregulation of genes like sterol regulatory element-binding protein 1C (SREBP-1C) and stearoyl-CoA desaturase 1 (SCD1), which are involved in liver lipid metabolism disorders (Jeyakumar and Vajreswari, 2022).

### Indirect pathway

**FIGURE 1:**
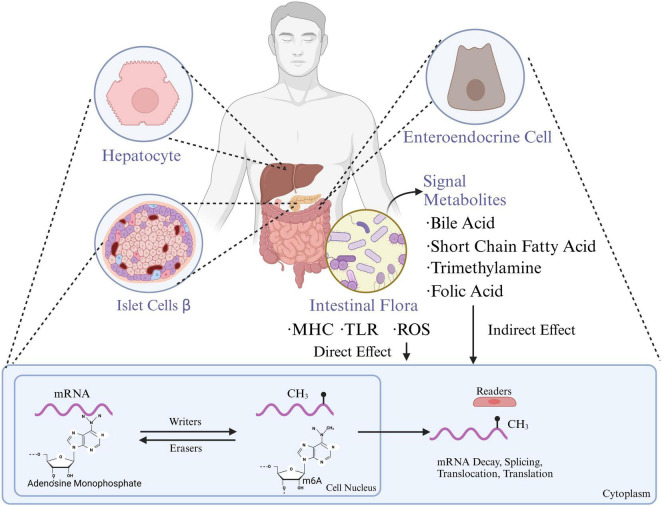
Gut microbiota influence the functions of hepatocytes, pancreatic islet β-cells, and enteroendocrine cells through metabolites which are also associated with mRNA methylation processes, impacting gene expression and metabolic regulation. MHC, major histocompatibility complex; TLR, toll-like receptors; ROS, reactive oxygen species; m6A, n6-methyladenosine (created with BioRender.com).

Gut microbiota metabolic products also play a broad role in regulating the host’s epigenome. As a “microbial endocrine organ,” the gut microbiota produces or transforms active substances such as SCFAs, secondary bile acids, various B vitamins, and S-adenosyl methionine (SAM), a major methyl donor (Monné et al., 2022). These metabolites indirectly induce epigenetic modifications. SAM is the primary methyl donor, while other gut-derived metabolites like methionine, choline, betaine, and folate contribute to the one-carbon cycle, which ultimately produces SAM. Vitamins B2, B6, and B12 from gut bacteria are also involved in the one-carbon cycle, aiding SAM synthesis. SAM is then absorbed into the bloodstream, supplying tissues and organs with the necessary methyl donors for m6A modifications. Other microbial metabolites, such as butyrate, can improve inflammation in granulosa cells in polycystic ovary syndrome by inhibiting METTL3-mediated m6A modifications of FOS-like antigen 2 (Shao et al., 2024). Meanwhile, secondary bile acids like deoxycholic acid can promote the dissociation of the METTL3-METTL14-WTAP complex, thus hindering the progression of gallbladder cancer. Hence, gut microbial metabolites are also important mediators in regulating host m6A modification levels (Figure 1).

## RNA, gut microbiota, and obesity

Obesity is a chronic disease characterized by excessive fat accumulation, with an increasing prevalence in recent years. It is also the primary risk factor for MAFLD (Gutiérrez-Cuevas et al., 2021). The causes of obesity are multifactorial, potentially involving interactions between genetic, behavioral, and social factors, and the widespread use of antibiotics may further exacerbate the condition. RNA plays a significant role in obesity. For example, a study using circRNA sequencing and bioinformatics analysis examined the expression of circRNAs in the subcutaneous adipose tissues of Large White and Laiwu pigs, identifying 70 upregulated and 205 downregulated circRNAs (Li et al., 2018). It was hypothesized that circRNAs may regulate adipogenesis and lipid metabolism. Overexpression of circ-SAMD4A has been associated with poor prognosis in obese individuals, as it regulates preadipocyte differentiation by acting as a miR-138-5p sponge (Liu et al., 2020), thereby increasing the expression of EZH2. Another study on visceral and subcutaneous fat deep sequencing revealed that various circRNAs are dynamically regulated during adipogenesis and obesity. Among them, circTshz2-1 and circArhgap5-2 were identified as key regulators of *in vitro* adipogenesis. What’s more, miRNA-193b-365 is aberrantly expressed in adipocyte differentiation and obesity states, and may be involved in adipocyte differentiation as well as the development of obesity (Goody and Pfeifer, 2019).

Since the discovery in 2006 that transferable obesity-related microbiota can induce weight gain in lean mice, subsequent epidemiological studies have demonstrated differences in gut microbiota between obese and lean individuals. At the species level, twin studies have shown that the abundance of SCFA producers, such as *Blautia* and *Roseburia* intestinalis, is associated with obesity, while butyrate producers like *Oscillospira* spp. (Ejtahed et al., 2020) and the methanogen *Methanobrevibacter smithii* are inversely correlated with obesity. Another genome-wide association study comparing lean and obese individuals found that in obese individuals, the abundance of *Phascolarctobacterium*, a glutamate-fermenting symbiont, was significantly lower and negatively correlated with serum glutamate levels (Álvarez-Mercado et al., 2019). In summary, several distinct gut microbiota have been linked to improved metabolic outcomes in mice, although further validation in humans is required. This effect is likely strain-specific and may depend on the presence of other supporting microorganisms, which can vary between individuals.

**FIGURE 2:**
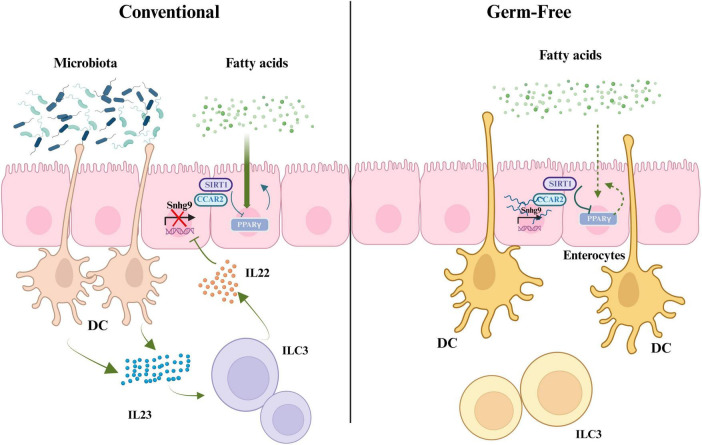
The role of gut microbiota in regulating fatty acid metabolism, PPARγ signaling, and IL22 production through interactions with immune cells. SIRT1, silent information regulator factor 2-related enzyme 1; CCAR2, cell cycle and apoptosis regulator 2; Snhg9, small nucleolar RNA host gene 9; PPARγ, peroxisome proliferator-activated receptor γ; DC, dendritic cell; IL23, interleukin-23; ILC3, group 3 innate lymphoid cells; IL22, interleukin-22 (created with BioRender.com).

The gut microbiota influences the transcription of long non-coding RNA (lncRNA) in intestinal epithelial cells, thereby affecting lipid metabolism. Transcriptome sequencing (RNA-seq) results indicate that gut microbiota suppresses the expression of lncRNA Snhg9 (Hashemi et al., 2024). Further research has found that gut microbiota indirectly inhibits the expression of lncRNA Snhg9 through myeloid cells and ILC3. lncRNA Snhg9 enhances the activity of SIRT1 by binding to its inhibitor CCAR2, which is a negative regulator of the lipid metabolism core regulator PPARγ (Zhang et al., 2020). Therefore, lncRNA Snhg9 inhibits lipid uptake through the CCAR2-SIRT1-PPARγ signaling axis, reducing body fat and preventing diet-induced obesity. These findings provide new strategies for treating metabolic diseases by targeting lncRNA Snhg9 and gut microbiota. However, further research is needed to determine which specific components or features of the gut microbiota can activate myeloid cell-ILC3 signaling and how the composition of gut microbiota affects the expression of Snhg9 and lipid metabolisms (Wang et al., 2023; Figure 2).

Metabolites from the gut microbiota also play a significant role in promoting obesity. The regulation of white adipose tissue (WAT) by gut microbiota is a critical process in maintaining metabolic health, and dysbiosis of gut microbiota can lead to the development of obesity (Sun et al., 2018). miRNAs are endogenous, non-coding single-stranded RNA molecules approximately 22 nucleotides long, involved in the degradation of target miRNA or inhibition of its translation, thereby negatively regulating post-transcriptional gene expression. The miR-181 family plays an important role in processes such as cell proliferation, apoptosis, and differentiation. Researchers have found that a high-fat diet activates miR-181 in the white adipose tissue of mice, subsequently leading to obesity and insulin resistance (Virtue et al., 2019).

In obese individuals, miR-181 levels are increased in white adipose tissue, while levels of indole are reduced in plasma, suggesting a potential correlation between the gut microbiota-miR-181 axis and human diseases (Eslam et al., 2022). Notably, researchers have observed dysregulation of miR-181 expression in white adipose tissue and the abundance of tryptophan-derived metabolites in a group of obese human children (Virtue et al., 2019). This indicates that the miR-181 family may represent a potential therapeutic target for regulating white adipose tissue function in the context of obesity.

Furthermore, dysregulation of the gut microbiota-miR-181 axis is essential for the development of obesity, insulin resistance, and inflammation in white adipose tissue in mice. In contrast to the AhR agonist Ficz, which improves glucose tolerance without affecting obesity, the administration of indole to high-density lipoprotein-fed mice can prevent obesity and impaired glucose tolerance (Polyzos et al., 2019). This suggests that, at least in mice, indole exerts beneficial effects through the AhR and miR-181. These findings highlight the crucial role of gut microbiota-derived metabolites in regulating miR-181 levels in white adipose tissue, indicating that host metabolism is adjusted in response to dietary and environmental changes (Virtue et al., 2019).

## RNA, gut microbiota and MAFLD

MAFLD is characterized by hepatic steatosis, excess fat accumulation in the liver, and metabolic liver dysfunction (Eslam et al., 2022). Obesity, a chronic metabolic disease closely linked to T2DM, cardiovascular diseases, and osteoarthritis, is the primary risk factor for MAFLD (Polyzos et al., 2019). There is a significant correlation between MAFLD and T2DM—over 70% of T2DM patients have MAFLD, and more than 20% of MAFLD patients either have or are at high risk of developing T2DM (Caussy et al., 2021).

The “gut-liver axis” plays a crucial role in linking the liver and intestines structurally and functionally, involving pathways such as the bile duct, portal vein, and systemic circulation (Rattanaprasert et al., 2019). The liver, receiving about 75% of its blood supply from the portal vein, is the first organ exposed to gut microbiota and their metabolites via the portal circulation (Albillos et al., 2020). A healthy gut barrier prevents the translocation of intestinal microbes and their toxic byproducts, while damage to the barrier can lead to microbial translocation, excessive immune activation, and the onset or progression of liver inflammation (Nicoletti et al., 2019). Consequently, the development of MAFLD can disrupt the gut microbiota, and abnormalities in the gut microbiome and its metabolites can reciprocally affect MAFLD progression (Jiang et al., 2020).

The human gut microbiota predominantly comprises Bacteroidetes, Firmicutes, Proteobacteria, and Actinobacteria, with Bacteroidetes and Firmicutes being the most dominant phyla (Gomaa, 2020; Jiang et al., 2020). Gut microbiota plays a significant role in regulating metabolism, immunity, and disease development, and it is closely associated with MAFLD (Alisi et al., 2024). Various barriers in the gut, including physical, biochemical, and immune defenses, limit the translocation of microbes and their byproducts (Ma et al., 2022). However, unhealthy dietary habits (e.g., high-sugar, high-fat diets, and overeating) can cause dysbiosis, leading to barrier dysfunction and immune homeostasis disruption (Jamar et al., 2021). On one hand, immune activation triggered by gut microbes and their metabolites can exacerbate liver damage, inflammation, and fibrosis, accelerating MAFLD progression (Liu et al., 2022). On the other hand, certain gut microbial metabolites, such as SCFAs and bile acids, can mitigate liver inflammation, oxidative stress, and steatosis (Visekruna and Luu, 2021). Compared to healthy individuals, MAFLD patients exhibit reduced gut microbial diversity and significant compositional alterations, with increased abundance of Gram-negative bacteria (e.g., *Bacteroides, Proteobacteria, and Enterobacteria*) and decreased levels of Firmicutes, especially SCFA-producing bacteria like *Lactobacillus* and *Ruminococcus* (Aron-Wisnewsky et al., 2020). This suggests that the gut microbiota may be a key factor in the pathology of MAFLD.

In recent studies, circRNAs have emerged as potential regulators in MAFLD. For instance, bioinformatics analysis by Ou et al. identified 450 dysregulated circRNAs in a MAFLD mouse model, with 298 upregulated and 152 downregulated circRNAs (Zeng et al., 2024). Guo et al. found that circRNA-0046367 is significantly downregulated in steatotic livers, with its suppression linked to miR-34a/PPARα interaction and lipid peroxidation (Yu et al., 2021). Additionally, circRNA-0046366 was shown to be downregulated during free fatty acid-induced hepatic steatosis, and its upregulation inhibited the miR-34a/PPARα interaction, reducing triglyceride levels and suppressing steatosis (Guo et al., 2018). These findings suggest that circRNA-0046367 and circRNA_0046366 may play critical roles in the pathogenesis of MAFLD (Yepmo et al., 2022). However, the exact mechanisms and roles of m6A-modified non-coding RNAs in MAFLD remain unclear.

**FIGURE 3:**
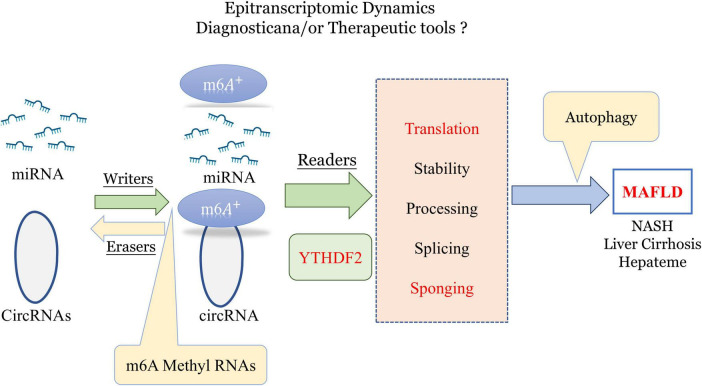
Role of epitranscriptomic m6A methylation in regulating miRNA and circRNA dynamics for potential diagnostic and therapeutic applications in MAFLD and liver diseases. m6A, n6-methyladenosine; YTHDF2, YTH N6-methyladenosine RNA-binding protein 2; MAFLD, metabolic-associated fatty liver disease (created with BioRender.com).

Research has demonstrated significant differences in m6A levels across various organs and tissues between germ-free and normal mice, indicating that gut microbiota strongly influences m6A mRNA modification (Jabs et al., 2020). m6A modifications may be crucial in forming the livers specific and complex ‘microenvironment (Pan et al., 2021). Initially, m6A modifications are regulated by methyltransferases (“writers”) and demethylases (“erasers”), with m6A+ binding proteins like YTH domain family 2 (YTHDF2), also known as “readers,” playing roles in RNA translation and autophagy regulation (He and He, 2021). Hence, m6A methylation in non-coding RNAs could serve as an important tool for diagnosing and treating various diseases, including MAFLD (Lin et al., 2020; Figure 3).

Recent studies have also linked nucleotide methylation to inflammation grading, lipid synthesis, and oxidative stress, underscoring its critical role in the progression of MAFLD. As a result, m6A modifications may be a key factor in the pathogenesis of MAFLD, providing new insights into disease mechanisms and potential therapeutic targets.

## RNA, gut microbiota, and metabolic heart disease

Metabolic heart disease encompasses a spectrum of cardiovascular disorders closely linked to metabolic dysregulation, primarily driven by disturbances in glucose and lipid metabolism (He and He, 2021). Common metabolic diseases such as diabetes mellitus and its complications, obesity, and fatty liver disease are recognized as significant contributors to this condition (Lin et al., 2020). Numerous studies have identified key risk factors for these metabolic disorders, including unhealthy dietary habits, sedentary lifestyles, and the aging process (Deprince et al., 2020). Notably, these risk factors are also prevalent among patients suffering from various cardiovascular diseases, including myocardial ischemia, atherosclerosis, and related complications (Yki-Järvinen, 2014). Recent projections indicate that by 2030, approximately 27 million individuals will be affected by hypertension, leading to a subsequent rise in coronary artery disease, stroke, and heart failure (Mozaffarian et al., 2008). This alarming trend underscores the high prevalence of metabolic heart disease within the population.

Given the intricate relationship between metabolic dysregulation and cardiovascular health, researchers have increasingly recognized “metabolic heart disease” as a critical term (Roth et al., 2020). This term encapsulates clusters of metabolic disturbances and their secondary effects on cardiovascular physiology (Bugger et al., 2022; Mohebi et al., 2022). In recent years, a wealth of research has emerged focused on these diseases, revealing the profound interplay between metabolic pathways and cardiovascular outcomes (Wang et al., 2022).

Key regulatory ncRNAs implicated in metabolic heart disease include miR-21, miR-126, miR-33, lncRNA H19, and lncRNA-ANRIL (Wishart, 2019). For instance, Surina et al. examined the pathological features of the left ventricle in a 5/6 nephrectomy rat model of chronic kidney disease (CKD), revealing an upregulation of miR-21-5p (Jusic et al., 2020). This upregulation was found to modulate the expression of various transcripts associated with fatty acid oxidation and glycolysis through the targeting of PPARα (Tudurachi et al., 2024). Further studies on H9C2 cells demonstrated that the overexpression of miR-21-5p diminished lipid content and lipid peroxidation, suggesting a regulatory role of miR-21-5p on the dependence of glycolytic and fatty acid oxidation pathways (Li et al., 2015).

Moreover, lncRNA-GAS5 and its target miR-21 have been implicated in regulating lipid levels, macrophage activity, T helper (Th) cell function, and vascular smooth muscle cell behavior, contributing to the progression of atherosclerosis and coronary heart disease (CHD) (Nasci et al., 2019). Investigations by Jiang and Du highlighted the correlation between the circulating levels of lnc-GAS5 and common biochemical markers, the severity of stenosis, and cytokine profiles in CHD patients, revealing a negative association with miR-21 levels. This suggests that lnc-GAS5 and miR-21 may serve as promising therapeutic targets for managing CHD.

In the pathophysiology of atherosclerosis, oxidized low-density lipoprotein (ox-LDL) plays a critical role in endothelial injury, oxidative stress, and inflammatory responses (Wang J. et al., 2019). miR-21 has been shown to mediate several pathophysiological mechanisms associated with atherosclerosis (Khatana et al., 2020). Pei et al. established an *in vitro* atherosclerosis model using ox-LDL to stimulate human umbilical vein endothelial cells (HUVECs) and vascular smooth muscle cells (VSMCs), investigating the potential functions of the CASC7/miR-21 axis in TLR4/NF-κB and PI3K/AKT signaling pathways (Canfrán-Duque et al., 2017). Their findings indicated that LncRNA-CASC7 regulates these pathways by inhibiting miR-21 expression, thereby influencing the pathological progression of ox-LDL-induced atherosclerosis (Pei et al., 2021).

Additionally, research has uncovered numerous circRNAs derived from genes associated with cardiovascular diseases, such as Ryr2, TTN, and DMD (Jalink et al., 2024). Notably, differential expression of circRNAs has been identified in both healthy individuals and those with cardiovascular conditions. A recent meta-analysis revealed 63 circRNAs with significantly abnormal expression in cardiovascular diseases, with 44 being upregulated and 19 downregulated (Bagheri Moghaddam et al., 2022). Among these, circCDKN2BAS and circMACF1 have been suggested as potential biomarkers for the diagnosis and treatment of cardiovascular diseases (Li et al., 2019). Furthermore, studies have indicated that the circRNA-MICRA, derived from zinc finger protein 609, exhibits lower levels in acute myocardial infarction patients, potentially serving as a biomarker for predicting left ventricular dysfunction (Li et al., 2019).

SCFAs, primarily produced through the fermentation of dietary fiber by gut microbiota, include acetate, propionate, and butyrate (Bayoumi et al., 2018). SCFAs play a vital role in modulating host metabolism and inflammatory responses and can act as signaling molecules influencing miRNA expression (Wang M. et al., 2019). For example, butyrate, as a histone deacetylase (HDAC) inhibitor, alters chromatin structure to regulate gene expression, including that of miRNAs (Yuan et al., 2019). Additionally, SCFAs can activate downstream signaling pathways via G protein-coupled receptors (such as GPR41 and GPR43), subsequently affecting miRNA expression, which may have significant implications for cardiovascular disease progression (Felice et al., 2015).

Moreover, trimethylamine N-oxide (TMAO), produced by gut microbiota through the metabolism of choline and carnitine, has been linked to an increased risk of cardiovascular diseases (Yang and Zhang, 2021). TMAO promotes the development of atherosclerosis by influencing cholesterol and bile acid metabolism (Zhen et al., 2023). Research indicates that TMAO can upregulate the expression of miR-33 in macrophages, a miRNA known to regulate cholesterol metabolism, thereby facilitating foam cell formation—a critical early event in atherosclerosis (Yang et al., 2022).

In conclusion, the exploration of metabolic heart disease encompasses the intricate interplay of metabolic dysregulation, gene expression regulation, and the influence of gut microbiota on cardiovascular health (Xue et al., 2022). These insights provide crucial foundations for understanding the underlying mechanisms and identifying potential therapeutic targets for addressing this pressing health concern (Santos et al., 2017; Tang et al., 2019).

## Conclusion and future perspectives

The interaction between RNAs, the gut microbiota and metabolic diseases is a rapidly evolving field that provides new insights into the mechanisms underlying these diseases. Current studies emphasize that the gut microbiota plays a key role in regulating host metabolism and influencing the expression of RNA molecules, especially non-coding RNAs such as miRNAs and lncRNAs. These RNA molecules are key regulators of various metabolic processes, including lipid metabolism, glucose homeostasis, and inflammatory responses, all of which are closely linked to the onset and progression of metabolic diseases such as T2DM, obesity, and MAFLD (Table 1).

**TABLE 1:** RNA and gut microbes in *in vivo* studies of metabolic diseases.

Disease	Cohort	RNA or gut microbiome crosstalk with disease	References
Diabetes	T1DM animal model	Pancreatic β cells miR-21 ?	(Lakhter et al., 2018)
Diabetes	33 patients with diabetic retinopathy and 30 healthy individuals	lncRNA-MEG3 regulates retinopathy angiogenesis through the VEGF signaling pathway	(Jia and Zhou, 2020)
Diabetes	High-glucose mouse models	CircRNA-15698? in mesenchymal cells, and TGF-β1 expression was up-regulated by miR-1851	(Ren and Wang, 2021)
Diabetes	/	CircRNA-HIPK3 blocks miR-30a function and promotes endothelial cell proliferation and vascular dysfunction in diabetic retinopathy	(Zhou and Kuang, 2021)
Diabetes	3 nondiabetic and 2 diabetic human retinas	circRNA-0005015 ? in patient body fluid samples	(Zaiou, 2020)
Diabetes	Patients with prediabetes and T2DM versus control	CircRNA-0054633? in the peripheral blood of the patient	(Pan et al., 2018)
Diabetes	345 patients with T2DM	butyrate-producing gut bacteria (*Clostridium* and *Butyricicoccus*)↓	(Wu et al., 2024)
Diabetes	GEO database	Butyrate expression was negatively correlated with miR-106a; Gut microbes can regulate miR-21 to act on the target gene Bcl-2 to induce apoptosis in β cells	(Gao and Zhao, 2020)
Diabetes	High-fat diet-induced mouse models	Hepatic triglyceride levels are related to the gut microbiota and the expression of liver miRNAs	(Wilson et al., 2017)
Obesity	Subcutaneous adipose tissues of Large White pig and Laiwu pig	70 up-regulated circRNAs and 205 down-regulated circRNAs	(Li et al., 2018)
Obesity	40 control and 60 obese individuals	CircSAMD4A acts as a miR-138-5p sponge, regulating the differentiation of preadipocyte	(Liu et al., 2020)
Obesity	Visceral and subcutaneous fat deep sequencing	CircTshz2-1 and circArhgap5-2 are key regulators of adipogenic *in vitro*; miRNA-193b-365 may be involved in adipocyte differentiation and the development of obesity	(Goody and Pfeifer, 2019)
Obesity	Systematic review	SCFA producers (*Blautia* and *Roseburia* intestinalis) is associated with obesity; Butyrate producers like *Oscillospira spp.* and the methanogen *Methanobrevibacter smithii* are inversely correlated with obesity	(Ejtahed et al., 2020)
Obesity	Genome-wide association study comparing lean and obese individuals	*Phascolarctobacterium*↓	(Álvarez-Mercado et al., 2019)
Obesity	High-fat diet induces mice	miR-181 is activated in WAT, leading to obesity and insulin resistance	(Virtue et al., 2019)
Obesity	19 non-obese children and 19 obese children	miR-181 is dysregulated in WAT	(Virtue et al., 2019)
MAFLD	MAFLD patients and healthy individuals	Bacteroides, Proteobacteria, and Enterobacteria?; Lactobacillus and Ruminococcus↓	(Aron-Wisnewsky et al., 2020)
MAFLD	NAFLD mouse model	450 dysregulated circRNAs	(Zeng et al., 2024)
Metabolic heart disease	/	63 circRNAs with significantly abnormal expression in cardiovascular diseases	(Bagheri Moghaddam et al., 2022)
Metabolic heart disease	Meta-analysis	CircCDKN2BAS and circMACF1 may be potential biomarkers for cardiovascular disease	(Li et al., 2019)
Metabolic heart disease	Meta-analysis	In patients with acute myocardial infarction, circRNA MICRA (derived from zinc finger protein 609) ↓	(Li et al., 2019)

The ability of the gut microbiota to regulate RNA expression through the production of metabolites such as SCFAs and TMAOs suggests that the microbiota-RNA axis is an important regulatory mechanism for metabolic health. These metabolites not only regulate gene expression, but also affect post-transcriptional processes such as mRNA stability and translation efficiency. For example, SCFAs can regulate miRNA expression by altering chromatin structure, whereas TMAO can promote atherosclerosis by regulating miR-33.

In addition, the role of the gut microbiota in epigenetic modifications, particularly m6A methylation, further strengthens the link between RNA, gut microbiota, and metabolic diseases. Studies have shown that dysbiosis in the gut microbiota leads to aberrant m6A modifications, which in turn affects the expression of key metabolic genes, leading to metabolic dysfunction. These findings highlight the potential of targeting RNA modifications and the gut microbiota to prevent and treat metabolic diseases.

The complexity of metabolic disease mechanisms is underscored by the inter-regulatory relationship between RNA and the gut microbiota. As researchers continue to unravel the intricate pathways involved, it is clear that both RNA and gut microbiota offer promising targets for therapeutic intervention. Future research should focus on further elucidating these interactions, particularly through large-scale, multicenter clinical trials, in order to identify biomarkers that contribute to the early diagnosis of metabolic diseases and personalized treatment strategies.

By delving into the interactions between the gut microbiota and RNA in the pathogenesis of metabolic diseases, we can gain valuable insights into the diagnosis and prevention of related metabolic diseases. Whether targeting the gut microbiota or RNA alone, or intervening in the communication pathways between the two, these approaches offer unprecedented potential for targeted therapies for metabolic diseases. However, due to differences in RNA secretion patterns-such as variations in composition and levels across race, gender, body fluids, and feces-as well as numerous factors affecting the stability of the gut microbiota, RNA expression and gut microbiota abundance can vary significantly between subjects, even within the same disease context.

Therefore, these factors must be considered comprehensively when investigating the interactions between RNA and gut microbiota in relation to pathological changes in the host. Large-scale, multicenter clinical trials should be conducted to further elucidate the regulatory relationship between RNA and the gut microbiota, moving from theoretical proof to more concrete evidence. These studies will help to determine whether the gut microbiota or RNA can serve as a biomarker for metabolic diseases, thereby contributing to the prevention, diagnosis, and monitoring of these diseases. Ultimately, this will provide a solid theoretical basis for the development of new targeted therapies.

In conclusion, the convergence of RNA biology and gut microbiota research opens new avenues for understanding the molecular basis of metabolic diseases. By advancing our knowledge in this area, we can develop innovative approaches to preventing and treating disease, ultimately improving outcomes for patients with these pervasive health problems.
